# A Randomized Clinical Study to Compare the Perioperative Analgesic Efficacy of Ultrasound-Guided Erector Spinae Plane Block Over Thoracic Epidural in Modified Radical Mastectomy

**DOI:** 10.7759/cureus.51103

**Published:** 2023-12-26

**Authors:** Deepshika R, Aruna Parameswari, Balasubramanian Venkitaraman, Mahesh Vakamudi, Akilandeswari Manickam

**Affiliations:** 1 Anesthesiology, Sri Ramachandra Institute of Higher Education and Research, Chennai, IND; 2 Surgical Oncology, Sri Ramachandra Institute of Higher Education and Research, Chennai, IND

**Keywords:** breast oncology, modified radical mastectomy (mrm), postoperative nausea vomiting, postoperative pain relief, ultrasound-guided erector spinae plane block (espb), thoracic epidural analgesia

## Abstract

Aim

This study aims to compare the effectiveness of ultrasound-guided erector spinae block (ESB) with thoracic epidural (TE) in managing postoperative pain among breast cancer (BC) surgery patients.

Methods

A total of 42 patients were enrolled and randomly divided into two groups, each comprising 21 participants. Primary endpoints assessed included intraoperative fentanyl consumption, postoperative pain scores, and the need for rescue analgesia. Secondary endpoints encompassed intraoperative hemodynamic changes and the incidence of postoperative nausea and vomiting (PONV).

Results

The study found no significant difference in intraoperative fentanyl requirement (p=0.62) or postoperative pain scores measured using numerical rating scores (NRS) throughout the 48-hour postoperative period. None of the patients in either group required rescue analgesia. Notably, there was a statistically significant difference in postoperative nausea and vomiting at the two-hour mark, favoring the erector spinae block. Both groups exhibited comparable hemodynamic changes during intraoperative monitoring.

Conclusions

Our investigation concludes that the ESF offers equivalent analgesic efficacy to the thoracic epidural during both surgery and the postoperative period without inducing any significant hemodynamic instability. Considering the lower complication rate associated with paraspinal blocks compared to neuraxial blocks, the ESB presents itself as a promising alternative method for effective pain relief in mastectomy procedures.

## Introduction

Breast cancer (BC) is a prevalent malignancy among women worldwide. In India, its incidence has risen remarkably, elevating its rank from fourth to first among common cancers. Recent trends reveal a higher occurrence in young women [1]. Surgical management is vital in non-metastatic cases, ranging from breast conservation to modified radical mastectomy based on various factors.

Effectively controlling acute pain curbs the surgical stress response, reducing opioid and anesthetic requirements. Thoracic epidural anesthesia (TEA) lessens cardiac and splanchnic sympathetic activity, impacting the perioperative function of vital organs. Surgery-related pain triggers heightened sympathetic activity, impacting hormonal and immune responses [2].

Approximately 60% of breast surgery patients encounter significant postoperative pain, underscoring limitations in conventional pain management and the need for improved approaches [3]. Multimodal analgesia involves various drugs targeting pain pathways (opioids, nonsteroidal anti-inflammatory drugs [NSAIDs], local anesthetics, and nerve blocks) to enhance pain control and reduce opioid usage and side effects. Regional anesthesia techniques localize local anesthetic administration, mitigating systemic effects. Truncal regional anesthesia before mastectomy significantly reduces immediate post-surgery pain scores and opioid consumption [4].

Historically, thoracic epidurals were the standard for truncal regional anesthesia. Erector spinae block (ESB), a newer technique introduced by Forero et al. in 2016 for thoracic neuropathic pain management, involves ultrasound-guided injection between erector spinae muscle and thoracic transverse process [5]. The injected agent induces a craniocaudal spread, creating a multi-dermatomal sensory block (blocking dorsal and ventral rami of spinal nerves) [6,7]. Compared to conventional neuraxial anesthesia, complications are minimal due to the injection's distance from the spinal cord and major blood vessels. Complications may include infection, pneumothorax, or block failure [8]. Erector spinae block effectively reduces acute postoperative pain, pain scores, and postoperative opioid consumption in mastectomy patients with breast cancer [9,10] while also delaying the need for rescue analgesia [9,11].

In this article, we detail our experience comparing the utilization of erector spinae block with the established gold standard regional anesthesia, thoracic epidural anesthesia, in patients undergoing modified radical mastectomy. 

## Materials and methods

Our study was conducted as a prospective, randomized clinical trial at our tertiary care center from March 2019 to February 2020, spanning 12 months. Prior to commencing the study, we secured approval from our institutional ethics committee (IEC/19/JUL/152/52). Our study was also registered in the Clinical Trial Registry, India (CTRI/2020/12/029736).

**Figure 1:**
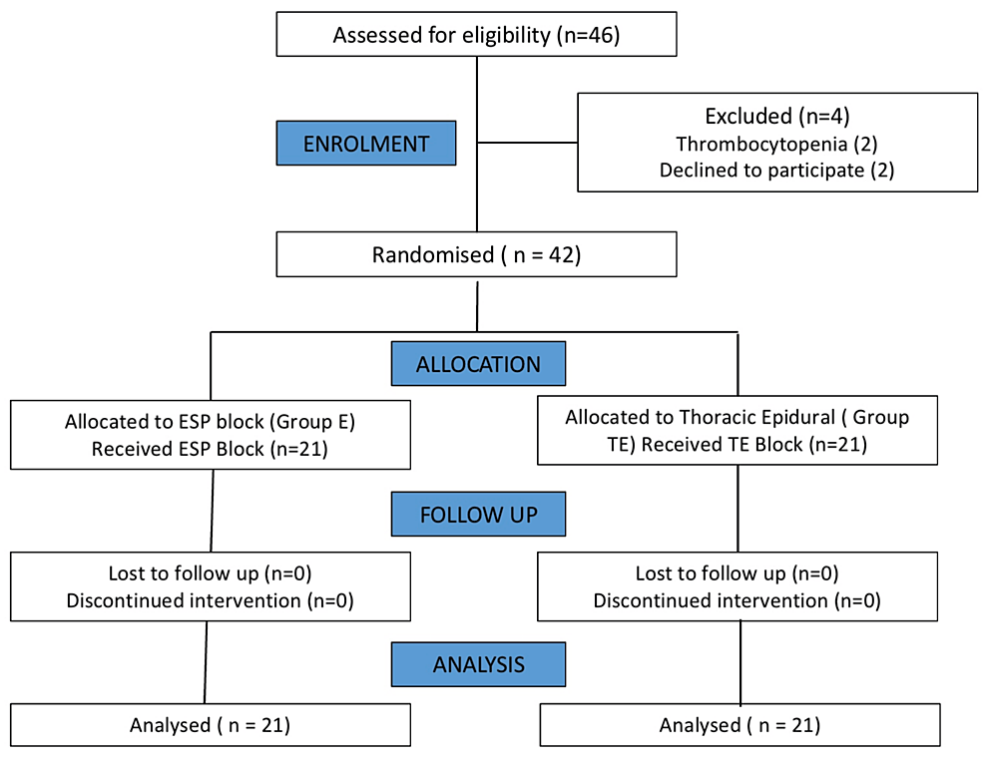
Consolidated Standards of Reporting Trials (CONSORT) diagram ESP - erector spinae plane

Breast cancer patients scheduled for unilateral modified radical mastectomy at our center, meeting inclusion criteria and providing informed written consent, were randomly assigned to one of two groups: erector spinae block (E) or thoracic epidural (TE), determined by a computer-generated random table. Out of the 46 eligible patients evaluated, four were excluded (two had thrombocytopenia, and two declined the procedure). The Consolidated Standards of Reporting Trials (CONSORT) in Figure 1 visually represents the study.

Inclusion and exclusion criteria

Patients satisfying the following criteria were included in the study: age group 20-70 years, with preoperative anesthesia evaluation showing American Society of Anesthesiologists (ASA) I-III, and those planned for unilateral modified radical mastectomy or breast conservation surgery.

Patients with anomalies of the vertebral column, with coagulation disorders or those on anticoagulants, those with thrombocytopenia (platelet count <100,000/mm^3^), with prior mastectomy surgery, with infection at the planned injection site, obese patients with BMI more than 35kg/m^2^, having an allergy to anesthetic drugs, those with failed blocks and those not consenting for the study were excluded.

Preoperative protocol

**Figure 2:**
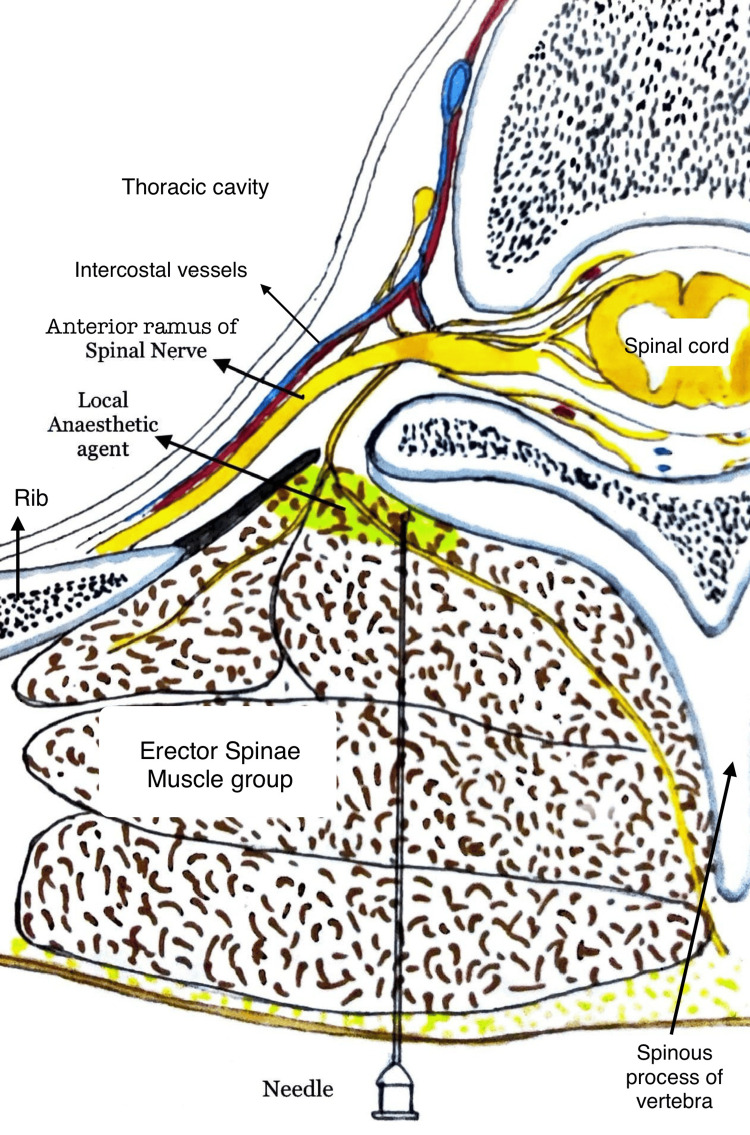
Erector spinae block Cross-sectional anatomy of erector spinae space with a graphical representation of the plane of injection of anesthetic agent. The image is created by the authors.

The procedures took place in the operating room before induction, with ultrasound guidance with the patient in a sitting or lateral decubitus position. Vital monitors, including heart rate, blood pressure, heart rhythm, and blood oxygen saturation, were affixed to patients, and their values were documented. For the erector spinae block, we administered an initial 10 ml of 0.25% bupivacaine solution via a 16 G Tuohy needle at the T5 level (Figure 2). Subsequently, an epidural catheter was inserted, directed cranially until approximately 5 cm remained within the erector spinae space. An additional 10 ml of 0.25% bupivacaine was delivered via the catheter. 

In the thoracic epidural arm, a thoracic epidural was performed at the T4-T5 level. An epidural catheter was placed in the epidural space using a 16 G Tuohy needle advanced to a depth of 4 cm. A test dose of 2% lignocaine with adrenaline was administered. Instances of dural puncture or intravascular injection were considered technique failures. Following the exclusion of these complications, epidural analgesia was initiated using 10 ml of 0.25% bupivacaine.

Intraoperative protocol

After completing the planned regional block technique, patients were repositioned to a supine position. Hemodynamics were monitored for five minutes before pre-oxygenation. Pre-oxygenation involved administering 100% O_2_ until the end-tidal oxygen level exceeded 90%. Intravenous induction was then carried out using a combination of propofol (2 mg/kg), fentanyl (2 mcg/kg), and vecuronium (0.1 mg/kg). Endotracheal intubation was performed under direct laryngoscopy. Anesthesia was maintained using sevoflurane, air, and oxygen to achieve a minimum alveolar concentration (MAC) of 1.2. A continuous infusion of bupivacaine (0.0625%) at 6 ml/hour was initiated through the epidural/erector spinae catheter 15 minutes after completing the block in both groups. The breast surgery began 15 minutes after the regional anesthesia procedure.

Baseline hemodynamic readings were recorded, and subsequent readings were taken at regular 10-minute intervals until the conclusion of the surgery. An increase of over 20% from preoperative levels indicated the need for analgesia, which was addressed by administering intravenous fentanyl at a dose of 0.5 mcg/kg of body weight. The total intraoperative opioid consumption was calculated and documented for both groups. Any complications, such as hypotension or bradycardia, were duly noted. About 30 minutes before the anticipated extubation time, intravenous paracetamol was administered at a dosage of 15 mg/kg alongside antiemetic prophylaxis (ondansetron 0.15 mg/kg). The inhalational anesthetic agent was discontinued upon completion of skin closure.

Upon surgery completion and the patient's achievement of sufficient tidal volume along with muscle power recovery, intravenous administration of injection neostigmine (0.05 mg/kg) and injection glycopyrrolate (0.01 mg/kg) was conducted to reverse neuromuscular blockade. The moment of extubation was designated as zero hours (zero hours post-extubation - 0 hrs PEX). The patient was positioned in a head-up posture and subsequently transferred to the post-anesthesia care unit (PACU).

Postoperative protocol

Patient parameters were continuously monitored within the PACU. In both groups E and TE, the infusion of 0.0625% inj. bupivacaine at 6 ml/hr was maintained for up to 48 hours post-extubation. After surgery, pain scores, heart rate, blood pressure, and postoperative vomiting were assessed at extubation and predetermined intervals. Pain intensity was gauged using a 10-point Numerical Rating Score (NRS). Postoperative analgesia included intravenous paracetamol at a dosage of 15 mg/kg every eight hours. In cases where the NRS score equaled or exceeded four, rescue analgesia was administered with inj. tramadol (1 mg/kg). If pain persisted 30 minutes following the first rescue analgesic, a second rescue analgesic, inj. ketorolac (30 mg intravenously), was given. Postoperative nausea and vomiting (PONV) were monitored at fixed intervals using a four-point categorical scale.

Statistical analysis

The sample size was calculated assuming the mean fentanyl consumption in the thoracic epidural block as 300 with a standard deviation of 70 and in the ESP block as 364.40 with a standard deviation of 70 [12]. The other parameters considered for sample size calculation were 80% power of study and 5% alpha error. The following formula was used for sample size calculation [13].

$N =( u+ v) ^2(\delta1 ^{2} +\delta2 ^{2}) / (\mu1-\mu0 )^{2}$

N - sample size; µ1-µ0 - difference between the means (300 and 364.4)

δ1 +δ2 - standard deviations (70 and 70)

u - one-sided percentage point of the normal distribution corresponding to 100% - the power. If the power is 80%, u is 0.84

v - percentage point of the normal distribution corresponding to the (two-sided) significance level. For a significance level of 5%, v is 1.96

As per the above-mentioned calculation, the required sample size was 42, with 21 in each group.

The primary endpoints studied were intraoperative opioid consumption, postoperative pain scores, and the need for rescue analgesia, while the secondary endpoints were intraoperative hemodynamic changes and PONV. Descriptive analysis was carried out using mean and standard deviation for quantitative variables, frequency and proportion for categorical variables.

The quantitative variables between the two groups were compared by comparing the mean values. The mean differences, along with their 95% CI, were calculated, and an independent sample t-test was used to assess statistical significance. The categorical outcomes were compared between two groups using cross-tabulations and comparison of percentages. The chi-square test and Fisher's exact test were used to test statistical significance. P value <0.05 was considered statistically significant. SPSS version 22 (IBM Inc., Armonk, US) was used for statistical analysis.

## Results

A total of 42 patients with unilateral breast cancer were randomized into two groups: the thoracic epidural (TE) arm and the erector spinae block (ESB) arm. Demographic data of the patients is presented in Table 1. The mean age was 51.16 years in the TE arm compared to 52.15 years in the E arm. BMI was similar in both arms (25.2 in TE compared to 26.4 in E arm). Upon analysis, both arms exhibited comparable demographic distributions (p>0.05).

**Table 1:** Demographic data of the patients

Parameters	Thoracic epidural group, n=21 (mean±SD)	Erector spinae block group, n=21 (mean±SD)
Age (years)	51.16±2.8	52.15±2.2
Weight (kg)	62.2±9.6	62.2±7.8
Height (cm)	157.2±5.2	153.6±7.9
BMI (kg/m^2^)	25.2±3.1	26.4±4.4

Intraoperative parameters are presented in Table 2. The mean surgery time was similar in both groups, with no statistically significant difference (p=0.492). The intraoperative opioid consumption (fentanyl) was also similar without any statistical difference. 

**Table 2:** Intraoperative parameters

Parameters	Thoracic epidural group (mean ±SD)	Erector spinae block group (mean ± SD)	p-value (2 tailed)
Duration of surgery (minutes)	109.6±12.9	112.2±11.0	0.492
Intraoperative fentanyl (microgram)	101.4±9.1	102.9±9.6	0.623

Pain scores, recorded on the NRS scale (0-10) at extubation, two, four, seven, 12, 24, and 48 hours, did not exhibit any statistical difference between the two groups (Table 3). None of the patients reported an NRS score of four or higher, and no patients required rescue analgesia at any point.

**Table 3:** Comparison of pain scores between the groups at various prespecified time intervals Group E - erector spinae block; group TE - thoracic epidural

Post-extubation time	Group	n	Mean	SD	Significance (2-tailed)
0 hours	Group E	21	0.38	0.67	0.306
	Group TE	21	0.19	0.51	
2 hours	Group E	21	0.52	0.81	0.06
	Group TE	21	0.14	0.36	
4 hours	Group E	21	0.33	0.73	0.099
	Group TE	21	0.05	0.22	
8 hours	Group E	21	0.43	0.87	0.176
	Group TE	21	0.14	0.36	
12 hours	Group E	21	0.57	1.12	0.119
	Group TE	21	0.14	0.48	
24 hours	Group E	21	0.33	0.97	0.312
	Group TE	21	0.1	0.44	
48 hours	Group E	21	0.29	0.64	0.055
	Group TE	21	0	0	

Regarding PONV, there was no notable distinction between the two groups in most readings. However, at the 2-hour mark, patients in the TE group demonstrated significantly increased PONV compared to the E group (p=0.014; see Table 4).

**Table 4:** Postoperative nausea and vomiting scores Postoperative nausea and vomiting scores were calculated at fixed intervals postoperatively in both groups, graded 0, 1, 2 and 3. TE - thoracic epidural group; E - erector spinae block group

Scores	0 hours		2 hours		4 hours		8 hours		12 hours		24 hours		48 hours	
Group	TE	E	TE	E	TE	E	TE	E	TE	E	TE	E	TE	E
0	14	17	14	19	20	20	20	21	20	21	20	21	21	21
1	6	3	6	1	1	1	1	0	1	0	1	0	0	0
2	1	0	1	1	0	0	0	0	0	0	0	0	0	0
3	0	1	0	0	0	0	0	0	0	0	0	0	0	0
Absent	14	17	14	19	20	20	20	21	20	21	20	21	21	21
Present	7	4	7	2	1	1	1	0	1	0	1	0	0	0
p-value	0.097		0.014		.147		.311		.311		.311		-	

Hemodynamic assessments, including heart rate, blood pressure changes, and mean arterial blood pressure, were conducted at ten-minute intervals throughout the surgery (Table 5). These assessments did not reveal any statistically significant differences between the two study groups.

**Table 5:** Hemodynamic assessment comparing the two groups Hemodynamic assessment comprising heart rate, systolic and diastolic blood pressure, and mean arterial blood pressure, recorded at 10-minute intervals. All pressures are recorded as millimeters of mercury (mm. Hg). Group E - erector spinae block; group TE - thoracic epidural

Time frame	Groups	Mean heart rate (mm. Hg)	Mean systolic blood pressure (mm. Hg)	Mean diastolic blood pressure (mm. Hg)	Mean arterial blood pressure (mm. Hg)
Preoperative	Group E	87	127.2	75.3	92.5
	Group TE	88.1	131.7	82.2	98.7
Pre-induction	Group E	90.6	143.2	82.1	102.3
	Group TE	93	143.4	86.2	105.3
0 min	Group E	88	137.8	80.3	99.5
	Group TE	88	134.9	77.9	96.9
5 min	Group E	83.9	115	70.1	85.1
	Group TE	80.6	123	73.7	90.1
10 min	Group E	84.5	116.6	73.3	87.7
	Group TE	77.4	121.1	75.2	90.5
20 min	Group E	81.5	114.4	73.2	86.9
	Group TE	76.2	116.3	71.3	86.3
30 min	Group E	79.8	117.6	72.3	87.4
	Group TE	74.9	113.4	69.9	84.4
40 min	Group E	80.7	120.5	75.4	90.4
	Group TE	75	115	71.6	86.1
50 min	Group E	78	122.3	74.2	90.3
	Group TE	73.4	117.4	72.7	87.6
60 min	Group E	78.8	121.8	75.1	90.7
	Group TE	76.3	119.2	74.4	89.4
70 min	Group E	77.3	122.2	74.6	90.5
	Group TE	76.1	120.7	73.7	89.3
80 min	Group E	79.5	124.5	75.2	91.6
	Group TE	78.3	121.7	74.9	90.5
90 min	Group E	79.3	124.5	75.8	92
	Group TE	82.1	125	77.5	93.4
110 min	Group E	79.5	120.3	73.3	89
	Group TE	80.1	119.9	76	90.6
130 min	Group E	80.3	119	72.9	88.2
	Group TE	77.5	119.1	74.3	89.3

The primary endpoints of the study included intraoperative opioid consumption, postoperative pain scores (NRS), and the need for rescue analgesia, which showed no statistically significant difference between the two groups. However, a notable distinction was observed in one of the secondary endpoints, specifically in the parameter of postoperative nausea and vomiting (PONV) at the two-hour mark, where the erector spinae block group exhibited a significant favorable difference.

## Discussion

Thoracic epidural anesthesia has traditionally been considered the gold standard for regional anesthesia following breast and thoracic procedures. It has been well-documented to contribute to a reduction in hospital stay [14], provide markedly improved pain relief compared to general anesthesia alone on days 0, 1, and 2 after surgery, and correlate with notably higher patient satisfaction levels [15].

In their randomized trial comparing erector spinae plane block to no additional procedure in breast cancer surgery, Wiech et al. found improved recovery (using QoR-40), along with statistically significant reductions in postoperative pain (p=0.012), pain scores, time to first opioid demand (p=0.014), and opioid consumption (p<0.01)[9]. Similar findings were documented by studies in the Indian subcontinent. Thiagarajan et al. demonstrated a significantly increased median time to first rescue analgesia [11], and Singh's study revealed a statistically significant reduction in postoperative opioid requirement in the erector spinae arm [10]. Hong et al. reported that ESB was significantly superior to the control group for patients undergoing mastectomy, showing median cumulative opioid consumption reduction, early postoperative pain level improvement, and decreased PONV occurrence [16]. Gurkan's study indicated reduced total opioid consumption with erector spinae block compared to the control group in breast cancer surgery patients [17].

In contrast to other published studies, our study employed an intermittent infusion via a catheter placed within the erector spinae space. In comparison to these studies, our patients in the erector spinae group experienced effective postoperative pain control, with none reporting an NRS score of four or higher and no need for rescue analgesia. Similar results were observed in Nair et al. series, where no rescue analgesia was required [18]. Furthermore, in our study, nausea and vomiting were significantly better managed in the erector spinae group when compared to the thoracic epidural group. This is notable, considering that other studies have also reported no significant occurrences of PONV [10,11,16].

We also conducted a comparison of intraoperative hemodynamics between the two groups to assess potential differences. Our findings demonstrated that both groups exhibited comparable and satisfactory hemodynamic control, a result consistent with the study conducted by Singh et al. [10]. Notably, no complications were observed within the erector spinae group, affirming the safety of this procedure. This aligns with similar outcomes reported in other studies [10,19]. Compared to conventional neuraxial blocks, erector spinae can be considered a safer alternative due to the absence of risks such as dural puncture, intrathecal injection, or epidural hematoma, as the entire procedure occurs outside the spinal canal. Moreover, erector spinae proves to be effective for surgical anesthesia as a standalone modality, particularly beneficial for high-risk patients.

In contrast to the paravertebral block, the erector spinae block boasts a shorter procedural duration and significantly delays the need for the first analgesia request during the postoperative period [20]. In a randomized trial, Eskandr et al. discovered that erector spinae block exhibited comparability to paravertebral block in terms of analgesia and morphine consumption, but the latter was linked to an incident of pneumothorax [21]. A meta-analysis by Weng et al. determined that erector spinae block surpassed systemic analgesia while maintaining comparability to paravertebral block [22]. A recent systematic review conducted by Guan et al. affirmed that erector spinae block led to notably reduced opioid consumption, decreased postoperative pain intensity, and fewer nerve-related complications [23].

Limitations

Our study employed an indwelling catheter for intermittent erector spinae block, as opposed to a single-shot block. Consequently, this necessitated a minimum hospital stay of 48 hours for the patients. We recruited 42 patients for the study based on the power of study calculation. However, a larger sample size may help us confirm our findings further. 

## Conclusions

Multiple randomized trials and meta-analyses have consistently established the superiority of erector spinae block compared to general anesthesia alone (without postoperative regional therapy) in breast cancer surgery patients. This superiority is evident in terms of reduced opioid consumption, improved pain scores, and a lower rate of complications. In our study, a pioneering effort, randomized erector spinae block against the established gold standard of thoracic epidural, the findings conclude that erector spinae block is equally effective, with no statistically significant differences. Moreover, the utilization of an indwelling catheter in erector spinae block has the potential to further curtail the need for additional opioids and subsequently reduce pain scores, as demonstrated in our study.
